# LncRNAs *LCETRL3* and *LCETRL4* at chromosome 4q12 diminish EGFR-TKIs efficiency in NSCLC through stabilizing TDP43 and EIF2S1

**DOI:** 10.1038/s41392-021-00847-2

**Published:** 2022-01-31

**Authors:** Yankang Li, Yue Shen, Mengyu Xie, Bowen Wang, Teng Wang, Jiajia Zeng, Hui Hua, Jinming Yu, Ming Yang

**Affiliations:** 1 Cheeloo College of Medicine, Shandong University, Jinan, Shandong Province 250112 China; 2 Shandong Provincial Key Laboratory of Radiation Oncology, Cancer Research Center, Shandong Cancer Hospital and Institute, Jinan, Shandong Province 250117 China; 3 Shandong University Cancer Center, Jinan, Shandong Province 250117 China; 4 Department of Radiation Oncology, Shandong Cancer Hospital and Institute, Shandong First Medical University and Shandong Academy of Medical Sciences, Jinan, Shandong Province 250117 China

**Keywords:** Lung cancer, Cancer therapy

## Abstract

Epidermal growth factor receptor (EGFR)-tyrosine kinase inhibitors (TKIs) are effective targeted therapy drugs for advanced non-small cell lung cancer (NSCLC) patients carrying sensitized *EGFR* mutations. The rapid development of EGFR-TKIs resistance represents a major clinical challenge for managing NSCLC. The chromosome 4q12 is the first genome-wide association study (GWAS)-reported locus associated with progression-free survival (PFS) of NSCLC patients treated with EGFR-TKIs. However, the biological significance of the noncoding transcripts at 4q12 in NSCLC remains elusive. In the present study, we identified two 4q12 long noncoding RNAs (lncRNAs) LCETRL3 and LCETRL4 which could significantly dimmish EGFR-TKIs efficiency. In line with their oncogenic role, evidently higher LCETRL3 and LCETRL4 levels were observed in NSCLC tissues as compared with normal specimens. Importantly, lncRNA LCETRL3 can interact with oncoprotein TDP43 and inhibit ubiquitination and degradation of TDP43. Similarly, lncRNA LCETRL4 can bind and stabilize oncoprotein EIF2S1 through reducing ubiquitin-proteasome degradation of EIF2S1. In particular, elevated levels of LCETRL3 or LCETRL4 in NSCLC cells resulted in stabilization of TDP43 or EIF2S1, increased levels of NOTCH1 or phosphorylated PDK1, activated AKT signaling and, thus, EGFR-TKIs resistance. Taken together, our data revealed a novel model that integrates two lncRNAs transcribed from the 4q12 locus into the regulation of EGFR-TKIs resistance in NSCLC. These findings shed new light on the importance of functionally annotating lncRNAs in the GWAS loci and provided insights to declare novel druggable targets, i.e., lncRNAs, which may unlock the therapeutic potential of EGFR-TKIs resistant NSCLC in the clinic.

## Introduction

Lung cancer is one of the most common malignancies in the world.^1^ Non-small cell lung cancer (NSCLC) accounts for about 85% of all incident lung cancer. The prognosis of NSCLC is still poor, with the 5-year survival rate of 15%.^1^ In clinics, the epidermal growth factor receptor (EGFR)-tyrosine kinase inhibitors (TKIs) have been proven to be effective for the treatment of advanced NSCLC patients carrying sensitized *EGFR* mutations including the exon 19 deletion or the L858R mutation.^2,3^ Gefitinib is the firstly approved orally bioavailable, competitive, reversible EGFR-TKI for NSCLC with *EGFR* mutations. The large randomized phase III IPASS trail indicated a beneficial effect on progression-free survival (PFS) in the NSCLC patients with *EGFR* mutation treated with gefitinib compared to patients treated with chemotherapy.^4,5^ Suppression of EGFR via Gefitinib impacts multiple signaling pathways involved in tumor proliferation such as the PI3K/AKT pathway.^4–6^ Despite the promising anti-NSCLC effects, almost all patients underwent EGFR-TKIs resistance sooner or later. Importantly, short PFS was observed in a large portion of advanced NSCLC patients after EGFR-TKIs therapy.^2,3^ Therefore, it is essential to declare biological mechanisms responsible for the development of EGFR-TKIs resistance in NSCLC.

Germline genetic variations, i.e., single-nucleotide polymorphisms (SNPs), could impact the pharmacokinetic and/or pharmacodynamic profiles of EGFR-TKIs in NSCLC patients.^7–12^ Interestingly, genome-wide association studies (GWASs) have identified the chromosome 4q12 locus significantly associated with PFS of advanced NSCLC patients treated with EGFR-TKIs in several independent cohorts.^7^ It has also been reported that *CLOCK*, *NMU* and *SRD5A3* in the 4q12 locus might be candidate protein-coding genes involved in NSCLC prognosis.^7^ However, it is still largely unclear how the 4q12 noncoding genes underly the biology of EGFR-TKIs resistance of NSCLC.

Long noncoding RNAs (lncRNAs) are a group of ncRNAs with a length greater than 200nt, which play a vital part in human cancers including NSCLC.^13–15^ Accumulating evidences elucidated that multiple lncRNAs contributed to the development of EGFR-TKIs resistance in NSCLC.^16–21^ For instance, lncRNA H19 conferred to EGFR-TKIs resistance via interacting and upregulating PKM2 and increasing levels of AKT phosphorylation in NSCLC.^18^ However, the role of lncRNAs transcribed from the 4q12 locus in NSCLC development and progression remains to be explored.

In the current study, we systematically examined five candidate lncRNAs at 4q12, which are named as Lung Cancer EGFR-TKIs Resistance Long noncoding RNA 1, 2, 3, 4, and 5 (LCETRL1, LCETRL2, LCETRL3, LCETRL4, and LCETRL5) (Supplementary Table 1). We identified lncRNAs LCETRL3 and LCETRL4 functioning as the novel pharmacogenomic regulators of EGFR-TKIs resistance. In NSCLC cells, high levels of LCETRL3 or LCETRL4 could evidently promote malignant proliferation and diminish EGFR-TKIs efficiency in vitro and in vivo. In support of this, lncRNAs LCETRL3 and LCETRL4 are highly expressed in NSCLC specimens. Importantly, LCETRL3 or LCETRL4 could stabilize oncoprotein TDP43 or EIF2S1, upregulate NOTCH1 expression or phosphorylated PDK1 levels, and, thus, lead to the activated AKT signaling of NSCLC cells.

## Results

### Elevated expression of lncRNAs LCETRL3 and LCETRL4 at chromosome 4q12 in NSCLC tissues

**Fig. 1:**
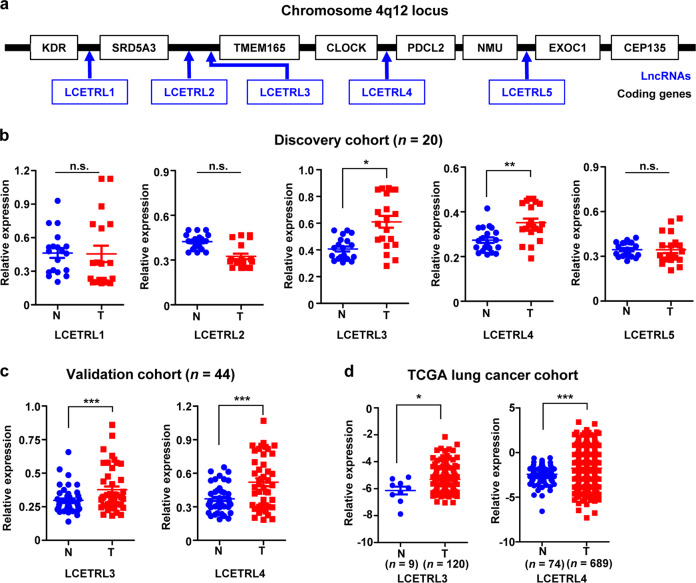
LCETRL3 and LCETRL4 are two significantly upregulated lncRNAs in NSCLC specimens. (**a**) Five candidate lncRNAs (LCETRL1, LCETRL2, LCETRL3, LCETRL4 and LCETRL5) locate at 4q12. (**b**) Relative expression of five candidate lncRNAs in paired NSCLC and normal tissues of Discovery cohort (*n* = 20). (**c, d**) Significantly elevated expression of lncRNAs LCETRL3 and LCETRL4 in NSCLC specimens of Validation cohort (*n* = 44) and TCGA lung cancer tissues compared to normal tissues. Two-tailed paired *t* test or two-tailed unpaired *t* test, n.s., not significant, ^*^P < 0.05, ^**^P < 0.01, ^***^P < 0.001

To reveal whether lncRNAs transcribed from the chromosome 4q12 locus are involved in NSCLC development, we firstly examined expression levels of five candidate lncRNAs (LCETRL1, LCETRL2, LCETRL3, LCETRL4, and LCETRL5) (Fig. 1a and Supplementary Table 1) in paired NSCLC and normal tissues of Discovery cohort (*n* = 20). We found that lncRNAs LCETRL3 and LCETRL4 showed significantly elevated expression in cancerous tissues compared to normal lung specimens (both *P* < 0.05) (Fig. 1b). Consistently, we found evidently increased levels of LCETRL3 and LCETRL4 in NSCLC specimens of Validation cohort (*n* = 44) (both *P* < 0.001) (Fig. 1c). We also investigated the aberrant expression of both lncRNAs in the TCGA lung cancer cohort and observed markedly increased LCETRL3 or LCETRL4 expression in NSCLC specimens compared to normal tissues (both *P* < 0.05) (Fig. 1d). The median follow-up time of all NSCLC patients in both the Discovery cohort and the Validation cohort was thirty months. After the last follow-up, we found that there were a few of NSCLC patients with disease progression. Two patients died and sixteen had disease progression. As a result, we examined the differences of the expression levels of LCETRL3 or LCETRL4 in NSCLC tissues between patients with disease progression and patients without disease progression (Supplementary Fig. 1a and b). We found that there was the significantly elevated expression of LCETRL3 or LCETRL4 in cases with disease progression compared to patients without disease progression (Supplementary Figure 1a and 1b). These data imply that lncRNAs LCETRL3 and LCETRL4 may act as novel oncogenes during NSCLC progression.

As shown in Fig. 1a, *LCETRL3* and *LCETRL4* are two adjacent lncRNA genes located in chromosome 4q12. Accumulating evidences indicated that lncRNAs may regulate adjacent gene(s) transcription and expression levels.^13–15,22^ First, we examined whether *LCETRL3* and *LCETRL4* regulate each other’s expression in NSCLC (Supplementary Fig. 1c and d). Silencing of lncRNA LCETRL3 did not impact levels of LCETRL4 in NSCLC cells (Supplementary Fig. 1c). Similarly, siRNAs of LCETRL4 did not influence the expression of LCETRL3 in NSCLC cells (Supplementary Fig. 1c). In support of this notion, no significant expression correlation between LCETRL3 and LCETRL4 was observed in NSCLC tissues of combined samples from the Discovery cohort and Validation cohort (*r*^2^ = 0.024, *P* = 0.238) (Supplementary Fig. 1d). Therefore, we speculated that LCETRL3 or LCETRL4 may independently play its part during NSCLC development.

### LCETRL3 and LCETRL4 promoted malignant proliferation of NSCLC in vitro and in vivo

**Fig. 2:**
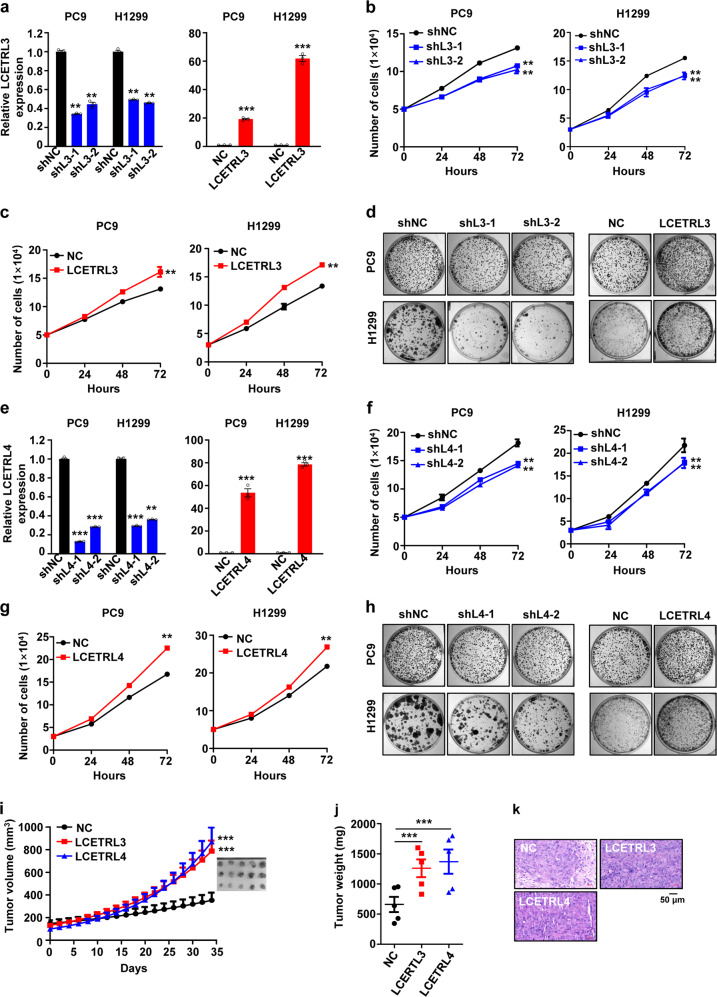
LncRNAs LCETRL3 and LCETRL4 suppressed malignant proliferation of NSCLC cells in vitro and in vivo. (**a**–**c**) Silencing of LCETRL3 with shRNAs inhibited proliferation of PC9 and H1299 cells; whereas overexpressed lncRNA LCETRL3 promoted proliferation of NSCLC cells. (d) LCETRL3 promoted clonogenicity of PC9 and H1299 cells. (**e**–**g**) Knocking-down of lncRNA LCETRL4 with shRNAs inhibited proliferation of PC9 and H1299 cells. On the contrary, overexpression of LCETRL4 promoted viability of NSCLC cells. (**h**) LCETRL4 stimulated colony formation of PC9 and H1299 cells. (**i**–**k**) In vivo growth of NSCLC xenografts was promoted by lncRNA LCETRL3 or LCETRL4. Two-tailed unpaired *t* test, ^**^P < 0.01, ^***^P < 0.001

We further investigated the involvement of lncRNAs LCETRL3 and LCETRL4 in NSCLC in vitro and in vivo. First, multiple NSCLC cell lines were generated via stably silencing LCETRL3 or LCETRL4 by shRNAs or forced-expressing the lncRNAs by plasmids. These NSCLC cells successfully transduced by lentivirus were selected by blasticidin or puromycin. In PC9 and H1299 cells stably expressing LCETRL3 shRNAs, there was significantly decreased expression of the lncRNA (shL3-1 or shL3-2 vs. shNC: *P* < 0.01) (Fig. 2a). Strikingly over-expressed LCETRL3 was found in PC9 and H1299 cells stalely expressing the LCETRL3 construct (LCETRL3 vs. NC: *P* < 0.001) (Fig. 2a). As shown in Fig. 2b and c, silencing of lncRNA LCETRL3 significantly inhibited proliferation of PC9 and H1299 cells (all *P* < 0.01); whereas, ectopic LCETRL3 expression markedly promoted the viability of NSCLC cells (both *P* < 0.01). Rescue assays indicated that over-expression of LCETRL3 could enhance cell proliferation of NSCLC cells after stable silencing of lncRNA LCETRL3 with shRNAs (Supplementary Fig. 2a). Consistently, knocking-down expression of LCETRL3 suppressed clone formation of PC9 and H1299 cells (Fig. 2d). NSCLC cells with overexpressed lncRNA LCETRL3 showed reinforced clonogenicity (Fig. 2d). We also found that LCETRL3 could profoundly promote the migration and invasion capability of NSCLC cells (Supplementary Figure 3). These data elucidated that lncRNA LCETRL3 acts as a novel oncogene during NSCLC pathogenesis.

There was evidently downregulated expression of lncRNA LCETRL4 in PC9 and H1299 cells stably expressing its shRNAs (shL4-1 or shL4-2 vs. shNC: *P* < 0.01) (Fig. 2e). On the contrary, markedly over-expression of lncRNA LCETRL4 was observed in both NSCLC cell lines stalely expressing the LCETRL4 construct (LCETRL4 vs. NC: *P* < 0.001) (Fig. 2e). It has been found that depleted expression of lncRNA LCETRL4 notably inhibited the proliferation of PC9 and H1299 cells (shL4-1 or shL4-2 vs. shNC: *P* < 0.01) (Fig. 2f). By contrast, the viability of NSCLC PC9 and H1299 cells was significantly promoted by ectopic LCETRL4 expression (LCETRL4 vs. NC: *P* < 0.01) (Fig. 2g). Moreover, over-expressed LCETRL4 enhance the growth of NSCLC cells after stable knocking-down of lncRNA LCETRL4 (shL4-1 or shL4-2) (Supplementary Fig. 2b). Colony formation assays indicated that the clonogenicity of NSCLC cells was obviously stimulated by lncRNA LCETRL4 (Fig. 2h). We also found that LCETRL4 could remarkably accelerate migration and invasion of PC9 and H1299 cells (Supplementary Fig. 4). Together, these data clarified the oncogenic functions of lncRNA LCETRL4 in NSCLC.

We next assessed the in vivo role of lncRNAs LCETRL3 and LCETRL4 using NSCLC xenografts. Importantly, xenografts stably over-expressing LCETRL3 grew much faster and showed an evident increase in tumor volume and tumor weight compared to the control xenografts (*P* < 0.001) (Fig. 2i–k). Similarly, growth of the NSCLC xenografts with stably forced expression of lncRNA LCETRL4 was significantly stimulated compared to the control tumors (*P* < 0.001) (Fig. 2i–k). On the contrary, stable silencing of LCETRL3 or LCETRL4 evidently inhibited growth of the NSCLC xenografts compared to the control xenografts (Supplementary Figure 5a and 5b). Collectively, these results suggested that LCETRL3 and LCETRL4 could promote the malignant proliferation of NSCLC in vivo.

### LCETRL3 and LCETRL4 significantly reduce gefitinib sensitivity

**Fig. 3:**
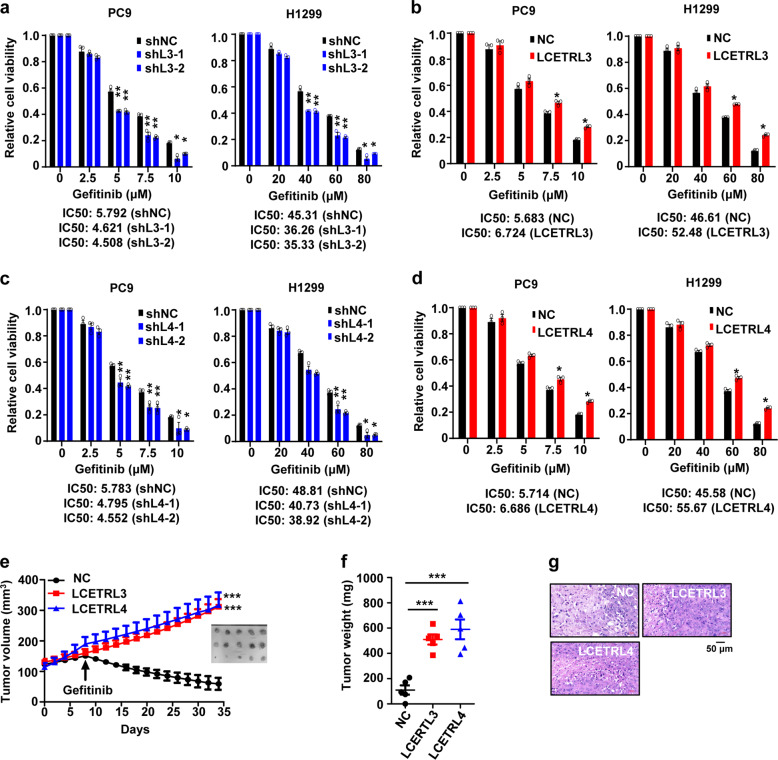
LncRNAs LCETRL3 and LCETRL4 significantly diminished the antiproliferation effects of gefitinib in vitro and in vivo. (**a, b**) LncRNA LCETRL3 evidently decreased the antiproliferation effects of gefitinib in PC9 and H1299 cells. (**c, d**) LncRNA LCETRL4 also obviously diminished the anticancer effects of gefitinib in NSCLC cells. (**e**–**g**) LCETRL3 or LCETRL4 significantly reduced efficiency of gefitinib using NSCLC xenografts in vivo. The arrow indicated the start time of gefitinib treatment. Two-tailed unpaired *t* test, ^*^P < 0.05, ^**^P < 0.01, ^***^P < 0.001

Considering *LCETRL3* and *LCETRL4* located at the GWAS-identified 4q12 locus significantly associated with PFS of the EGFR-TKIs-treated NSCLC patients, we further evaluated how these lncRNAs impact the gefitinib sensitivity of NSCLC cells. As shown in Fig. 3a, the proliferation of the *LCETRL3*-KD NSCLC cells (shL3-1 or shL3-2) treated with gefitinib were notably retarded compared to the control cells treated with gefitinib (*P* < 0.05). In line with this, overexpression of LCETRL3 markedly promoted the proliferation of PC9 or H1299 cells treated with gefitinib (*P* < 0.05) (Fig. 3b). Similarly, silencing of lncRNA LCETRL4 expression notably strengthened the anticancer activities of gefitinib (*P* < 0.05) (Fig. 3c). Ectopic LCETRL4 expression strikingly stimulated cell viability of NSCLC cells treated with gefitinib (*P* < 0.05) (Fig. 3d). We also evaluated the in vivo impacts of lncRNA LCETRL3 or LCETRL4 on gefitinib sensitivity using NSCLC xenografts. We found that gefitinib could obviously suppress the proliferation of NSCLC tumors in mice. However, stably ectopic expression of LCETRL3 or LCETRL4 evidently diminishes gefitinib efficiency in NSCLC xenografts (Fig. 3e–g).

### LncRNA LCETRL3 suppressed TDP43 degradation via the ubiquitin-proteasome pathway and activated the NOTCH1-PTEN-AKT signaling

**Fig. 4:**
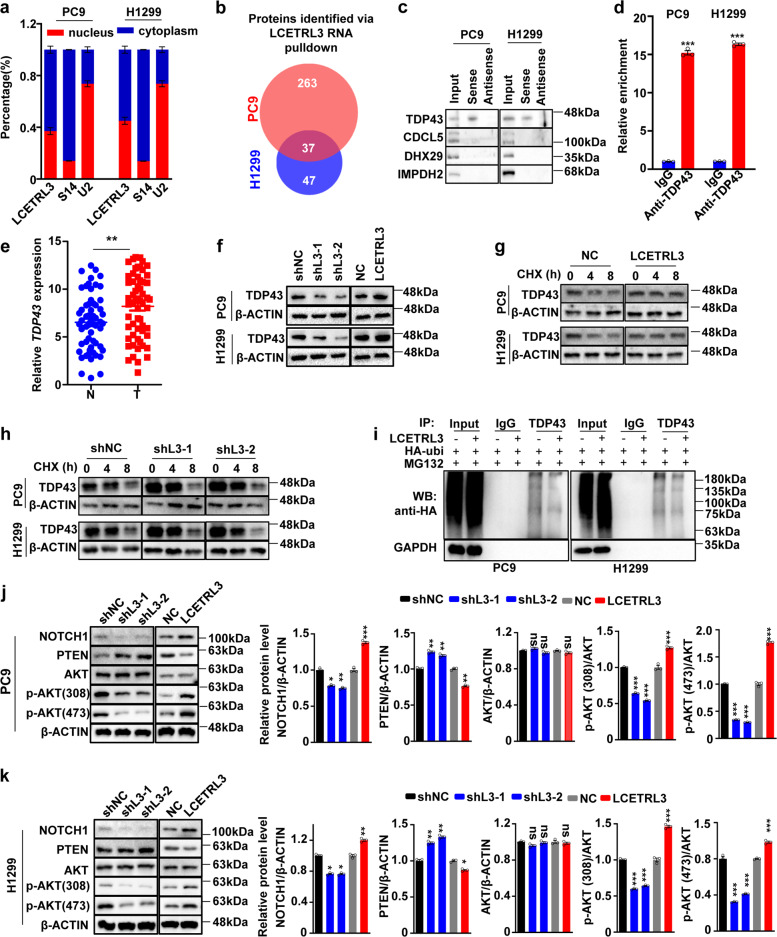
LncRNA LCETRL3 interacted with TDP43, prevented TDP43 degradation and activated the AKT signaling. (**a**) Cellular location of LCETRL3 in NSCLC PC9 and H1299 cells. (**b**) LCETRL3 pulldown proteins were identified by mass spectrometry in PC9 and H1299 cells. (**c**) Western blot validation of TDP43 pulled down by lncRNA LCETRL3. (**d**) LCETRL3 could be precipitated with antibody against TDP43 as compared with the IgG control in PC9 and H1299 cells. Relative enrichment (means ± SD) represents lncRNA LCETRL3 levels precipitated with TDP43 relative to an input control from three independent experiments. (**e**) There was markedly increased *TDP43* expression in NSCLC tissues compared to normal tissues of the combined samples in the Discovery and Validation cohorts (*n* = 64). (**f**) The TDP43 protein levels in PC9 and H1299 cells after either silencing of LCETRL3 or over-expressing LCETRL3 without any treatments. (**g, h**) Western blot analyses of TDP43 protein levels in PC9 and H1299 cells with stable over-expressed or silenced LCETRL3 after treatment of cycloheximide (CHX). (**i**) Western blot analyses of the ubiquitination of TDP43 in PC9 and H1299 cells that stabilized either silenced LCETRL3 or over-expressed LCETRL3. (**j, k**) LCETRL3 obviously upregulated NOTCH1 expression, suppressed PTEN expression and promoted phosphorylation of AKT in PC9 cells (**j**) and H1299 cells (**k**). Two-tailed unpaired *t* test, ns, not significant, ^*^P < 0.05, ^**^P < 0.01, ^***^P < 0.001

To explore how LCETRL3 and LCETRL4 controlling NSCLC development and gefitinib sensitivity, we firstly detected the impacts of both lncRNAs on the expression of adjacent protein-coding genes at 4q12 (Supplementary Figure 6). However, there were no evident expression changes of adjacent protein-coding genes at 4q12 (*SRD5A3*, *KDR*, *TMEM165*, *CLOCK*, *PDCL2*, *NMU*, *EXOC1*, and *CEP135*) after silencing of lncRNA LCETRL3 (Supplementary Figure 6a) or LCETRL4 (Supplementary Figure 6b). Considering the crucial functions of lncRNAs as protein-binding scaffolds during carcinogenesis,^13–15,23^ we hypothesized that lncRNAs LCETRL3 and LCETRL4 may bind certain proteins, disturb their downstream signaling and, thus, accelerate the proliferation of NSCLC cells. To test the hypothesis, we firstly examined the cellular localization of lncRNA LCETRL3 in NSCLC cells and found that there was almost equal LCETRL3 in the nucleus or cytoplasm (Fig. 4a). RNA pulldown assays plus mass spectrometry proteomics with the PC9 or H1299 cell extracts indicated that there were 270 proteins or 84 proteins pulled down by lncRNA LCETRL3 (Fig. 4b). Among these proteins, thirty-seven LCETRL3-pulled down proteins were observed in both NSCLC cell lines (Fig. 4b). We found that TDP43 is the 1^st^ most abundant protein among all proteins identified through mass spectrometry (Supplementary Table 2). Importantly, TDP43 was successfully validated through independent RNA pulldown assays and Western blot in PC9 or H1299 cells (Fig. 4c). Nevertheless, other three candidate proteins (CDCL5, DHX29 and IMPDH2) could not be verified (Fig. 4c). As compared with the IgG control, there was obvious enrichment of lncRNA LCETRL3 in RNA-protein complexes precipitated with the anti-TDP43 antibody in both NSCLC cell lines (*P* < 0.001) (Fig. 4d).

As shown in Fig. 4e, significantly elevated *TDP43* expression in cancerous tissues was observed compared to normal tissues in combined samples from the Discovery cohort and Validation cohort (*P* < 0.01), indicating the oncogenic nature of *TDP43* in lung cancer. Intriguingly, the knockdown of LCETRL3 markedly downregulated TDP43 protein levels in cells (Fig. 4f). Conversely, over-expressed LCETRL3 increased TDP43 protein expression in cells (Fig. 4f). Rescue assays indicated that over-expression of TDP43 could promote cell viability of NSCLC cells after stable silencing of lncRNA LCETRL3 with shRNAs (Supplementary Fig. 7a). To explore whether lncRNA LCETRL3 may impact TDP43 protein degradation, we detected TDP43 expression in cells treated with the protein synthesis inhibitor CHX. In ectopic LCETRL3 NSCLC cells, the protein levels of TDP43 reduced slower compared to the control cells (Fig. 4g). However, the protein levels of TDP43 reduced much faster in *LCETRL3*-KD PC9 and H1299 cells compared to the control cells (Fig. 4h), suggesting that LCETRL3 may suppress the proteasome degradation of TDP43 protein. We next investigated if LCETRL3-regulated degradation of TDP43 was mediated by its ubiquitination. After PC9 or H1299 cells were transfected with HA-ubi, endogenous TDP43 protein was immunoprecipitated. Evidently weakened ubiquitin signals of TDP43 were observed in NSCLC cells with stably ectopic LCETRL3 expression compared with those in the control cells (Fig. 4i). These results elucidated that that lncRNA LCETRL3 promotes TDP43 stabilization via the ubiquitin-proteasome system.

Multiple lines of evidence demonstrated that TDP43 is a DNA/RNA-binding protein involved in RNA metabolism including the *NOTCH1* mRNA.^24–26^ Indeed, silencing of LCETRL3 induced significantly downregulated NOTCH1 expression in both the PC9 and H1299 cells (shL3-1 and shL3-2 vs. shNC; *P* < 0.05) (Fig. 4j and k). In line with these data, overexpression of LCETRL3 upregulated NOTCH1 expression in NSCLC cells (LCETRL3 vs. NC; *P* < 0.01) (Fig. 4j and k). NOTCH1 activates AKT through repressing PTEN expression in multiple malignancies including NSCLC.^27–29^ Importantly, knocking-down of LCETRL3 increased PTEN expression and, thus, reduced levels of phosphorylated AKT (T450 and S473) in cells; whereas ectopic LCETRL3 obviously inhibited PTEN expression and enhanced AKT phosphorylation in NSCLC cells (Fig. 4j and k). Together, these data indicated that lncRNA LCETRL3 may stabilize TDP43 protein and activate the NOTCH1-PTEN-AKT signaling in NSCLC.

### LncRNA LCETRL4 activated the AKT signaling via stabilizing EIF2S1

**Fig. 5:**
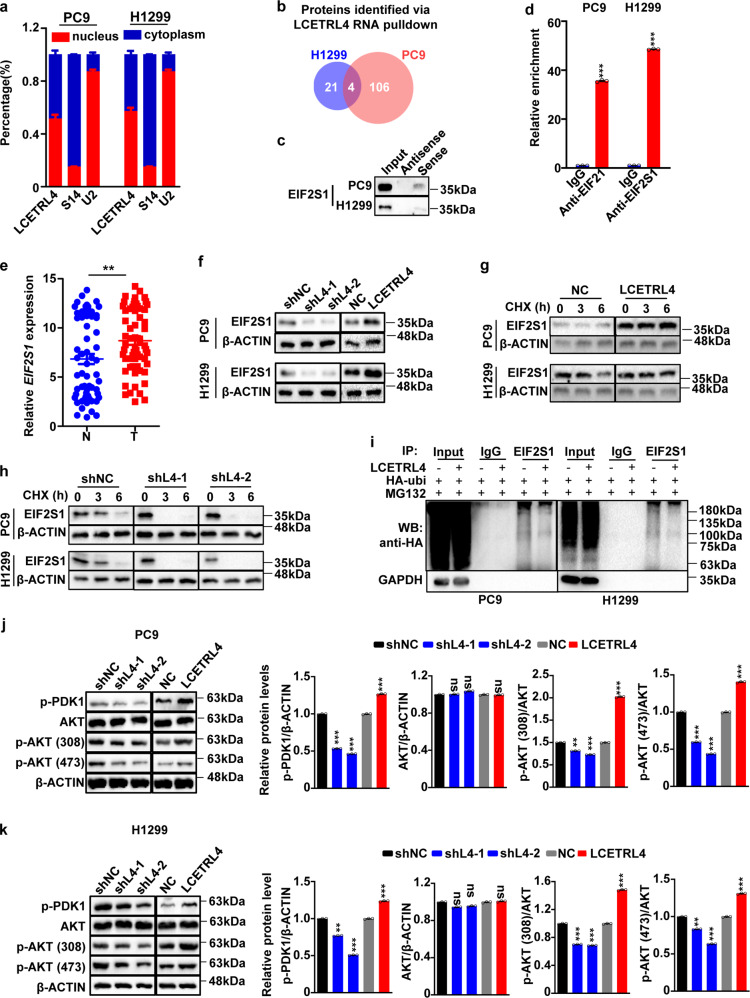
LncRNA LCETRL4 stabilized EIF2S1 protein and activated the AKT signaling. (**a**) Cellular location of LCETRL4 in PC9 and H1299 cells. (**b**) RNA pulldown assays plus mass spectrometry proteomics indicated that there were four proteins pulled down by lncRNA LCETRL4 in PC9 or H1299 cells. (**c**) Western blot validation of EIF2S1 pulled down by lncRNA LCETRL4. (**d**) LCETRL4 could be precipitated with antibody against EIF2S1 as compared with the IgG control in PC9 and H1299 cells. Relative enrichment (means ± SD) represents lncRNA LCETRL4 levels precipitated with EIF2S1 relative to an input control from three independent experiments. (**e**) There was evidently evaluated *EIF2S1* expression in NSCLC tissues compared to normal tissues of the combined samples in Discovery and Validation cohorts (*n* = 64). (**f**) The EIF2S1 protein levels in PC9 and H1299 cells after either silencing of LCETRL4 or over-expressing LCETRL4 without any treatments. (**g, h**) Western blot analyses of EIF2S1 protein levels in PC9 and H1299 cells with stable over-expressed or silenced LCETRL4 after treatment of CHX. (**i**) Western blot analyses of the ubiquitination of EIF2S1 in PC9 and H1299 cells that stabilized either silenced LCETRL4 or over-expressed LCETRL4. (**j, k**) LCETRL4 significantly enhanced phosphorylation of PDK and AKT in PC9 cells (**j**) and H1299 cells (**k**). Two-tailed unpaired *t* test, ns, not significant, ^**^P < 0.01, ^***^P < 0.001

Subcellular fractionation assays demonstrated that lncRNA LCETRL4 exists nearly in the same amount in the nuclear fraction or the cytoplasm fraction of NSCLC cells (Fig. 5a). After performing RNA pulldown assays plus mass spectrometry proteomics, we identified 110 proteins (PC9 cells) or 25 proteins (H1299 cells) pulled down by lncRNA LCETRL4 (Fig. 5b). Among four LCETRL4-pulled down proteins recognized in both cell lines, EIF2S1 is the 1^st^ most abundant protein (Fig. 5b and Supplementary Table 3). Consistently, we successfully verified EIF2S1 through Western blot using samples of independent RNA pulldown assays in PC9 or H1299 cells (Fig. 5c). RIP assays indicated that lncRNA LCETRL4 could be markedly enriched in the RNA-protein complexes precipitated with the EIF2S1 antibody as compared with the IgG control in NSCLC cells (*P* < 0.001) (Fig. 5d).

There was remarkably increased *EIF2S1* expression in cancerous tissues compared with that in normal tissues in combined samples from the Discovery cohort and Validation cohort (*P* < 0.01) (Fig. 5e). Interestingly, silencing of LCETRL4 evidently suppressed EIF2S1 protein expression in NSCLC cells (Fig. 5f), indicating that LCETRL4 might be involved in the regulation of the proteasome degradation of EIF2S1. Rescue assays indicated that over-expression of EIF2S1 could promote cell growth of the *LCETRL4*-KD NSCLC cells (Supplementary Fig. 7b). To further confirm whether lncRNA LCETRL4 impacts EIF2S1 degradation, we examined EIF2S1 expression in PC9 and H1299 cells treated with CHX. In *LCETRL4*-OE PC9 or H1299 cells, LCETRL4 diminished down-regulation of EIF2S1 protein after CHX treatment (Fig. 5g). Conversely, we observed that the EIF2S1 protein levels decreased much faster in the *LCETRL4*-KD cells compared to the control cells (Fig. 5h). To test if LCETRL4-dependent degradation of EIF2S1 was mediated by its ubiquitination, we examined ubiquitination levels of endogenous EIF2S1 immunoprecipitated in PC9 or H1299 cells transfected with HA-ubi. Obviously decreased ubiquitin signals of EIF2S1 protein were detected in the *LCETRL4*-OE cells compared to the control cells (Fig. 5i). Taken together, these data revealed that that lncRNA LCETRL4 could stabilize EIF2S1 via repressing its ubiquitination and degradation through proteasome.

EIF2S1 is a subunit of the translation initiation factor EIF2 complex.^30,31^ Interestingly, mouse embryonic fibroblasts with the *Eif2s1* mutation grew 50% slower and showed reduced Pdk1-Akt-mTOR signaling compared to wild-type cells.^31^ We hypothesized that high levels of LCETRL4 may stabilize EIF2S1, reinforce phosphorylation of PDK1 and, thus, promote activation of the AKT signaling in NSCLC. To test it, we detected levels of these proteins and the phosphorylated ones in PC9 and H1299 cells (Fig. 5j, k). Silencing of LCETRL4 evidently reduced phosphorylation levels of PDK1 (S241) and AKT (T450 and S473); whereas ectopic LCETRL4 improved levels of phosphorylated PDK1 (S241) and phosphorylated AKT (T450 and S473) in cells (Fig. 5j, k). However, dysregulated LCETRL4 did not influence total AKT protein expression in cells (Fig. 5j, k). These results demonstrated that high levels of LCETRL4 could stabilize EIF2S1 and activate the PDK1-AKT signaling in NSCLC.

## Discussion

**Fig. 6:**
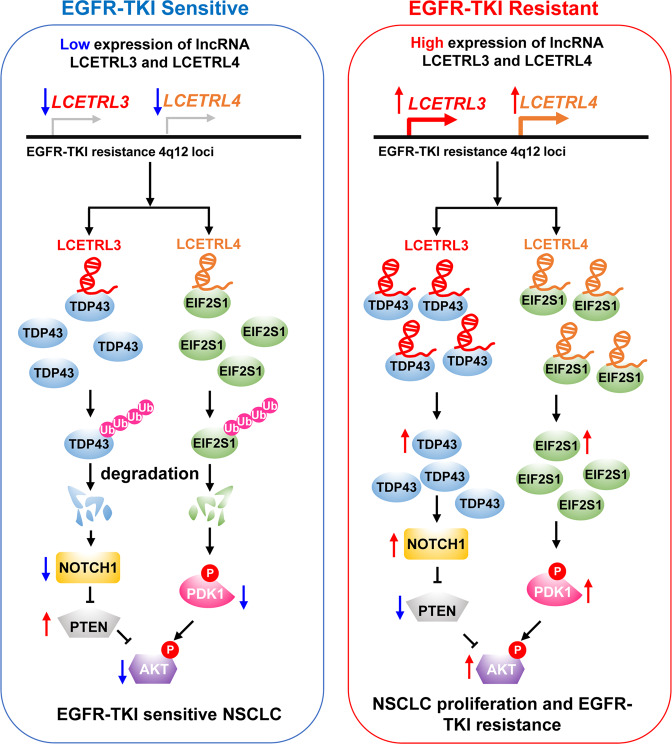
Graphical representation of the oncogenic functions of lncRNA LCETRL3 or LCETRL4 in EGFR-TKIs treatment of NSCLC. In EGFR-TKIs sensitive cells, low levels of lncRNA LCETRL3 or LCETRL4 cause deactivation of the AKT signaling in NSCLC. However, high levels of LCETRL3 or LCETRL4 could stabilize and upregulate oncoproteins TDP43 or EIF2S1, increase levels of NOTCH1 or phosphorylated PDK1, activate the AKT signaling and, thus, result in EGFR-TKIs resistance of NSCLC cells

The chromosome 4q12 is the first GWAS-identified locus associated with PFS of advanced NSCLC patients treated with EGFR-TKIs.^7^ For protein-coding genes at 4q12, *NMU* which encodes a GPCR ligand was known to be involved in NSCLC progression.^32,33^ However, the biological significance of the noncoding transcripts at 4q12 remains elusive in NSCLC. In the current study, we identified two 4q12 lncRNAs LCETRL3 and LCETRL4 which could dimmish the efficiency of EGFR-TKIs treatments. In line with their oncogenic nature, evidently higher LCETRL3 and LCETRL4 levels were observed in NSCLC tissues as compared with normal specimens. Importantly, lncRNA LCETRL3 can interact with oncoprotein TDP43 and inhibit ubiquitination and degradation of TDP43. Similarly, lncRNA LCETRL4 can bind and stabilize oncoprotein EIF2S1 through reducing ubiquitin-proteasome degradation of EIF2S1. In particular, elevated levels of LCETRL3 or LCETRL4 in NSCLC resulted in high expression of TDP43 or EIF2S1, increased levels of NOTCH1 or phosphorylated PDK1, and, thus, the activated AKT signaling (Fig. 6). Collectively, our data present new clues for interpretation of the genetic differences of individuals receiving EGFR-TKIs therapy from a new perspective and highlight the importance of protein ubiquitination modulated by lncRNAs in NSCLC.^34^

Multiple lncRNAs have been shown to be implicated in malignant development and EGFR-TKIs resistance in NSCLC.^16–21^ For example, tumor suppressor lncRNA GAS5 might play a role in gefitinib resistance which could be reversed by overexpressing GAS5.^16^ Similarly, downregulated expression of lncRNA H19 or HOTAIR contributed to resistance to EGFR-TKIs and low levels of H19 or HOTAIR were significantly correlated with shorter PFS in NSCLC patients treated with EGFR-TKIs.^18,20^ On the contrary, several oncogenic lncRNAs (SNHG15, LOC554202 and CRNDE) could promote EGFR-TKIs resistance through different mechanisms. Huang et al found that lncRNA SNHG15 can act as a sponge of miR-451 and facilitate expression of MDR-1 which posing proproliferation, promigration, and antiapoptosis effects on gefitinib-resistant NSCLC cells.^19^ LncRNA LOC554202 upregulated miR-31 expression, repressed RASA1 and FIH-1 expression, and thus, at least partially activated the RAF-MEK-ERK and PI3K-AKT signaling pathways in NSCLC with acquired resistance to gefitinib.^17^ Inhibition of lncRNA CRNDE activated the eIF4A3/MUC1/EGFR signaling and apoptotic activities, and restored sensitivity of NSCLC cells to EGFR-TKIs.^21^ In line with these reports, we for the first time found that lncRNAs *LCETRL3* and *LCETRL4* at the 4q12 locus are novel oncogenes and reduce the antiproliferation effects of gefitinib on NSCLC cells.

We revealed that lncRNA LCETRL3 can interact with TDP43 protein and suppress degradation of TDP43, an RNA-binding protein that participates in multiple steps of RNA metabolism, including transcription, splicing, and transport of mRNA, as well as miRNA metabolism.^24^ TDP43 has been shown to promote growth and metastasis of NSCLC, triple-negative breast cancer, neuroblastoma, hepatocellular carcinoma and melanoma.^35–40^ Consistently, we found that TDP43 was significantly upregulated in NSCLC tissues. It has been reported that Lys-48 and Lys-63 are two major ubiquitination sites of TDP43 and both sites are linked with polyubiquitin chains.^41,42^ However, TDP-43 of the Lys-48-linked polyubiquitin chains undergoes ubiquitin proteasomal-mediated degradation, while the TDP-43 with Lys-63-linked polyubiquitin chains might be removed via autophagy.^43^ In NSCLC, lncRNA LCETRL3 may mainly block ubiquitination of Lys-48 through binding to TDP43, inhibit proteasomal-mediated degradation of TDP43 and, thus, stabilize oncoprotein TDP43. Our study also demonstrated that increased expression of TDP43 controlled by lncRNA LCETRL3 could stabilize the *NOTCH1* mRNA, elevate NOTCH1 expression, downregulate PTEN expression and activate AKT in NSCLC cells, which thereby accelerating tumor growth and reduce EGFR-TKIs efficiency.

In eukaryotic cells, the aberrant accumulation of unfolded or misfolded proteins at the endoplasmic reticulum (ER) induces ER stress, which is emerging as a possible driver of human cancers and contributes to resistance to chemotherapy, target therapy and immunotherapy.^44–46^ EIF2S1, also known as EIF2α, plays a key role in the PERK-EIF2α signaling, which is one of three major ER stress branches. PERK phosphorylates eIF2α and reduces the overall frequency of mRNA translation initiation under ER stress.^44–46^ Of note, mouse embryonic fibroblasts carrying the *Eif2s1* mutation showed reduced activation of the Pdk1-Akt signaling.^31^ In line with this, we observed interactions between LCETRL4 and EIF2S1 lead to elevated EIF2S1 expression, deceased levels of phosphorylated PDK1 and stimulated AKT signaling.

In summary, we revealed a novel model that integrates two lncRNAs transcribed from the 4q12 locus into the regulation of oncogenic programs and EGFR-TKIs resistance in NSCLC. These findings shed new light on the importance of functionally annotating lncRNAs in the GWAS loci and provided insights to declare EGFR-TKIs resistance mechanisms developed by cancer cells. Considering that rapid development of resistance to targeted therapy drugs represents a major challenge for managing NSCLC, we believe that identification of novel druggable targets, i.e., lncRNAs, may unlock the therapeutic potential of NSCLC in the clinic.

## Material and Methods

### Patients and tissue specimens

There are two NSCLC patient cohorts (Discovery cohort and Validation cohort) in this study. In the Discovery cohort, twenty NSCLC patients were recruited at Shandong Cancer Hospital and Institute (Jinan, Shandong Province, China) between October 2016 and June 2017. In the Validation cohort, forty-four NSCLC patients were recruited at Shandong Cancer Hospital and Institute between July 2017 and November 2019. All stage I or II NSCLC patients received curative surgical resection. For the stage, IIIa NSCLC patients, surgical resection of the primary tumor site was performed to alleviate symptoms if patients were tolerable for the surgery. Chemotherapy and/or radiotherapy were given to these stage IIIa NSCLC patients after surgery. Fresh cancerous specimens and normal lung tissues were sampled from these patients. Normal lung tissues were obtained at least 2 cm away from the NSCLC border. All cases were Han Chinese. The detailed characteristics of all patients were shown in Supplementary Table 4. This study was approved by the institutional review board of Shandong Cancer Hospital and Institute. At recruitment, written informed consent was obtained from each subject. The methods were carried out in accordance with the approved guidelines.

### Quantitative reverse transcription PCR (RT-qPCR)

Total RNA from cultured cells or tissue specimens was isolated with Trizol reagent (Invitrogen, 94402). Each RNA sample was treated with DNase I (RNase-free) to remove genomic DNA (Thermo Fisher, 18068015). Each RNA sample was then reverse-transcribed into cDNAs using PrimeScript^TM^ RT Master Mix (TaKaRa, RR036A). Relative RNA levels of candidate genes were calculated by using the 2^−ΔΔCt^ method. Indicated primers are listed in Supplementary Table 5. Each sample was examined at least in triplicate. PCR product specificity was confirmed by melting-curve analyses.

### Cell culture

Human NSCLC H1299 cell line was obtained from the Shanghai Cell Collection, Chinese Academy of Sciences. Human NSCLC PC9 cells was purchased from JENNIO Biological Technology. HEK293T cells were kindly provided by Dr. Yunshan Wang (Jinan Central Hospital, Shandong Province, China). Cells were cultured in Dulbecco’s modified Eagle’s medium (DMEM) (Gibco, C11995500BT) with 10% fetal bovine serum (FBS; Gibco, 1347575). Cells were maintained at 37 °C in a 5% CO_2_ incubator and periodically tested and found to be negative for mycoplasma.

### RNA interference assays

Small interference RNAs (siRNAs) of LCETRL3 (siL3-1 and siL3-2), LCETRL4 (siL4-1 and siL4-2) as well as the negative control RNA (NC RNA) were ordered from Genepharma (Shanghai, China) (Supplementary Table 6). As reported previously,^10,12^ all small RNAs were transfected to PC9 and H1299 cells using the INTERFERin reagent (Polyplus, 409-10).

### The expression and shRNA constructs of LCETRL3 or LCETRL4

The full-length lncRNA *LCETRL3* or *LCETRL4* cDNA was directly synthesized by Genewiz (Suzhou, Jiangsu Province, China) and cloned after the CMV promoter of the pCDH-CMV-MCS-EF1-Puro vector. The plasmid was named as LCETRL3 or LCETRL4. Two shRNA hairpins targeting human *LCETRL3* (shLCETRL3-1 or shLCETRL3-2) or *LCETRL4* (shLCETRL4-1 or shLCETRL4-2) or the control shRNA (Supplementary Table 7) were cloned into the pLKO.1 vector. The resultant plasmids were designated shLCETRL3-1, shLCETRL3-2, shLCETRL4-1, shLCETRL4-2, or shNC. All aforementioned plasmids were sequenced to confirm the orientation and integrity.

### Lentiviral transduction

The lentivirus LCETRL3, LCETRL4, shLCETRL3-1, shLCETRL3-2, shLCETRL4-1, shLCETRL4-2 plasmid was co-transfected into HEK293T cells with the psPAX2 (Addgene, #12260) and pMD2.G (Addgene, #12259) plasmids using the jetPRIME reagent (Polyplus, 114-07) as reported previously.^23,47^ At 48 h and 72 h following transfection, recombinant lentiviral particles of LCETRL3, LCETRL4, shLCETRL3-1, shLCETRL3-2, shLCETRL4-1, shLCETRL4-2 and their controls (NC or shNC) in the viral supernatants were collected. Human PC9 and H1299 cells were infected with the LCETRL3, LCETRL4, shLCETRL3-1, shLCETRL3-2, shLCETRL4-1, shLCETRL4-2, NC or shNC viral supernatants. NSCLC cells were then selected using 2 μg/mL puromycin. In these lentiviral transducted cells, the expression levels of LCETRL3 or LCETRL4 were examined by RT-qPCR.

### Cell proliferation and gefitinib drug sensitivity analyses

For cell proliferation assays, a total of 5 × 10^4^ of PC9 cells or 3 × 10^4^ H1299 cells after lentiviral transduction of *LCETRL3*-knockdown (KD), *LCETRL4*-KD, *LCETRL3*-overexpression (OE) or *LCETRL4*-OE were seeded in 12-well plates. NSCLC cells were harvested and counted at 24, 48, and 72 h after seeding. For gefitinib drug sensitivity analyses, a total of 7500 cells of the stably transfected PC9 and H1299 cells were seeded per well in 96-well plates. Gefitinib diluted in DMSO was added to each well to achieve the desired final concentrations (PC9: 0 μmol/L, 2.5 μmol/L, 5 μmol/L, 7.5 μmol/L, and 10 μmol/L; H1299: 0 μmol/L, 20 μmol/L, 40 μmol/L, 60 μmol/L, and 80 μmol/L) as described previously.^9^ After cells were incubated with gefitinib for 48 h, 10 μL of 0.5 mg/mL MTT labeling reagents (Sigma, #88417, Temecula, CA, USA) were added to each well. One hundred microliters of DMSO were added to each well after the cells were incubated with MTT for 4 h in a cell culture incubator. Absorbance in each well was measured at 570 nm using a microplate reader.

### Colony formation assays

The stably *LCETRL3*-KD, *LCETRL4*-KD, *LCETRL3*-OE, or *LCETRL4*-OE PC9 (2,000 cells per well) were seeded into a 6-well cell culture plate. When colonies were visible after 8 days, cells were washed with cold PBS twice and fixed with the fixation fluid (methanol:acetic acid = 3:1). The cells were dyed with crystal violet and the number of the NSCLC colony in each well was counted. A total of 500 stably *LCETRL3*-KD and *LCETRL4*-KD H1299 cells or 2000 stably *LCETRL3*-OE and *LCETRL4*-OE H1299 cells per well were seeded into a 6-well cell culture plate. For the *LCETRL3*-KD and *LCETRL4*-KD H1299 cells, the NSCLC colony number was counted after 16 days. The colony number of the *LCETRL3*-OE and *LCETRL4*-OE H1299 cells was counted after 8 days.

### NSCLC xenografts

To evaluate the in vivo role of LCETRL3 and LCETRL4, we inoculated subcutaneously a total of 6 × 10^6^ *LCETRL3*-OE, *LCETRL4*-OE or control (NC) PC9 cells into fossa axillaries of five-week-old female nude BALB/c mice (Vital River Laboratory, Beijing, China) (*n* = 5 per group). Tumor growth was measured every two days after tumor volumes equaled to 100 mm^3^. To examine the involvement of LCETRL3 and LCETRL4 in gefitinib drug sensitivity in vivo, similar *LCETRL3*-OE, *LCETRL4*-OE or NC PC9 xenografts (*n* = 5 per group) were obtained as described previously.^23,47^ At the 8th day (tumor volumes equaled to or exceeded 200 mm^3^), 15 mg/kg gefitinib per mouse was given daily by intragastric administration. Frozen section and H&E staining were processed as described previously.^23,47^ All procedures involving mice were approved by the institutional Animal Care Committee of Shandong Cancer Hospital and Institute.

### Transwell assays

The transwell chambers (pore 8 mm, Corning) were firstly coated with 60 μL BD Biosciences Matrigel (1:20 dilution) for 12 h in a 5% CO_2_ incubator. PC9 or H1299 cells were added to upper transwell chambers, with a total of 650 μL medium with 10% FBS in the lower wells. After 48 h, cells migrated to the lower wells through pores were stained with 0.2% crystal violet solution and counted.

### Subcellular fractionation

The cytosolic and nuclear fractions of PC9 or H1299 cells were separately isolated using the nuclear/cytoplasmic Isolation Kit (Biovision, #P0027, China) according to the manufacturer’s instructions. The relative levels of LCETRL3 or LCETRL4 in cytosolic or nuclear fractions were detected by qRT-PCR.

### RNA pulldown

RNA pulldown was performed following the standard protocol as reported previously.^23,48–50^ To prepare the plasmid construct template for in vitro RNA synthesis, *LCETRL3* or *LCETRL4* was subcloned into pcDNA3.1 with inserted T7 promoter before and after the cloning site. After these constructs were linearized, lncRNAs were transcribed with T7 RNA polymerase (MEGAscript T7 Transcript Kit, Thermo fisher, AM1330) and purified with the RNeasy minikit (Qiagen, #74104, Germany). Pierce™ RNA 3’ End Desthiobiotinylation Kit (Thermo fisher, 20163) was used to biotinylate sense and antisense LCETRL3 or LCETRL4 RNAs. These RNAs were then incubated with PC9 or H1299 protein extracts at 4 °C for 1 h. Proteins bound on the streptavidin magnetic beads were eluted with the Elution Buffer of Pierce™ Magnetic RNA-Protein Pull-Down Kit (Thermo, 20164). The recovered proteins were then analyzed by liquid chromatography-tandem mass spectrometry (LS-MS/MS) (Hoogen Biotech Co., Shanghai, China) and Western Blot. MaxQuant software (version 1.5.3.30) with the UniProtKB human database (uniport *Homo sapiens* 188441_20200326) was utilized to analyze mass spectra.

### RNA immunoprecipitation (RNA-IP)

Magna RIP RNA-Binding Protein Immunoprecipitation Kit (Millipore, 17–700) with the EIF2S1 and TDP43 antibodies or IgG Isotype-control were used for RNA-IP assays as reported previously.^23,47^ The EIF2S1-RNA or TDP43-RNA complexes were recovered by Dynabeads® Protein G beads (Invitrogen, Lot#00929981). LncRNA LCETRL3 or LCETRL4 levels in the precipitates were measured by RT-qPCR.

### Western blot

Western blot was performed as described previously.^23,51^ After extracted from culture cells, total proteins of NSCLC cells were separated with the SDS-PAGE gel and transferred to a polyvinylidene fluoride (PVDF) membrane (Millipore, ISEQ00010). The PVDF membrane was then incubated with antibodies against TDP43, NOTCH1, EIF2S1, AKT, p-AKT (308), p-AKT (473), PTEN, p-PDK1, GAPDH or β-ACTIN (Supplementary Table 8) overnight at 4 °C. Proteins were visualized with ECL Western Blotting Substrate (Pierce, 32106).

### Turnover assays

The stably *LCETRL3*-KD, *LCETRL4*-KD, *LCETRL3*-OE or *LCETRL4*-OE PC9 and H1299 were seeded into 6-well plates and then cultured for 24 h. To stop *de novo* protein synthesis, cells were incubated with CHX (# 66-81-9, Merck, US) at a final concentration of 200 μg/mL. At the indicated times after CHX treatments, the PC9 and H1299 cells were harvested. Western blot was performed to examine the TDP43, EIF2S1 and β-ACTIN protein levels in NSCLC cells.

### Ubiquitination assays

Ubiquitination assays were carried out in PC9 and H1299 cells which were transfected with the pcDNA3.1-HA-ubiquitin (HA-ubi) plasmid as reported previously.^23^ At 36 h after transfection, NSCLC cells were incubated with 50 μg/mL MG132 for 6 h and then lysed with the ice-cold RIPA buffer (Beyotime, P0013C). To isolate ubiquitinated TDP43 or EIF2S1, proteins in the cell lysate were immunoprecipitated with anti-TDP43 or anti-EIF2S1 antibodies and then detected with the anti-HA antibody by Western blot.

### Statistics

The 2-tailed unpaired Student’s *t* test or paired Student’s *t* test was used to examine differences between two groups. The criterion of statistical significance was a *P* value of less than 0.05. All analyses were performed with GraphPad Prism (Version 5.0, GraphPad Software, Inc.).

## Supplementary information

Supplementary files

## Data Availability

The additional data collected during this study are available from the corresponding author upon reasonable request.
