# Supplementary material for: LncRNAs LCETRL3 and LCETRL4 at chromosome 4q12 diminish EGFR-TKIs efficiency in NSCLC through stabilizing TDP43 and EIF2S1
Source: Signal Transduct Target Ther. 2022 Jan 31;7:30. doi: 10.1038/s41392-021-00847-2 (PMC8801511; doi:10.1038/s41392-021-00847-2)
Supplement: Supplementary file 1 — Supplementary files [file 41392_2021_847_MOESM1_ESM.docx]

Supplementary Materials for

**LncRNAs *LCETRL3* and *LCETRL4* at chromosome 4q12 diminish EGFR-TKIs efficiency in NSCLC through stabilizing TDP43 and EIF2S1**

Yankang Li^1^; Yue Shen^1^; Mengyu Xie^1^; Bowen Wang^1^; Teng Wang^1^; Jiajia Zeng^2^; Hui Hua^2^; Jinming Yu^3,4,^*; Ming Yang^1,2,3,^*^,#^

**Authors’ affiliations:** ^1^Cheeloo College of Medicine, Shandong University, Jinan, Shandong Province, 250112, China; ^2^Shandong Provincial Key Laboratory of Radiation Oncology, Cancer Research Center, Shandong Cancer Hospital and Institute, Jinan, Shandong Province, 250117, China; ^3^Shandong University Cancer Center, Jinan, Shandong Province, 250117, China; ^4^Department of Radiation Oncology, Shandong Cancer Hospital and Institute, Shandong First Medical University and Shandong Academy of Medical Sciences, Jinan, Shandong Province, 250117, China.

***Correspondence to:** Ming Yang, Ph.D., Professor, Shandong Provincial Key Laboratory of Radiation Oncology, Cancer Research Center, Shandong Cancer Hospital and Institute, Jinan 250117, Shandong Province, China. Tel & Fax: 86531-67626536; E-mail: aaryoung@yeah.net.

or

Jinming Yu, M.D., Professor, Department of Radiation Oncology, Shandong Cancer Hospital and Institute, Jinan 250117, Shandong Province, China. Tel & Fax: 86531-67626971; E-mail: sdyujinming@126.com.

**This PDF file includes:**

Figures. S1 to S7

Tables S1 to S8


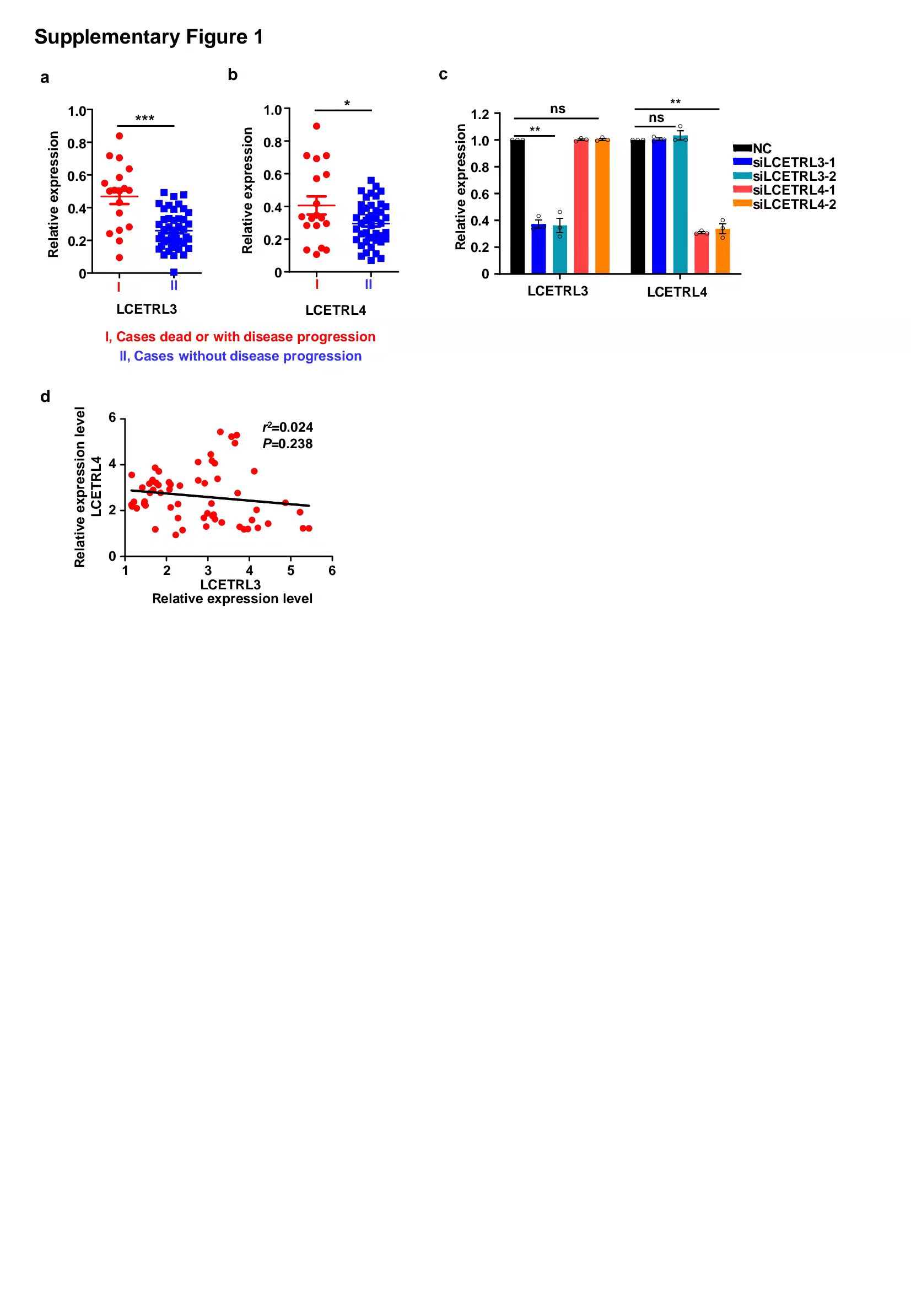


**Supplementary Figure 1. There is no mutual regulation and expression correlation between lncRNAs LCETRL3 and LCETRL4 in NSCLC.** (a, b) Relationship between therapeutic effect and lncRNA LCETRL3 or LCETRL4 expression level. (c) Silencing of lncRNA LCETRL3 or LCETRL4 did not affect expression of LCETRL4 or LCETRL3 in NSCLC PC9 cells. (d) No expression correlation between lncRNA LCETRL3 and LCETRL4 existed in NSCLC tissues.


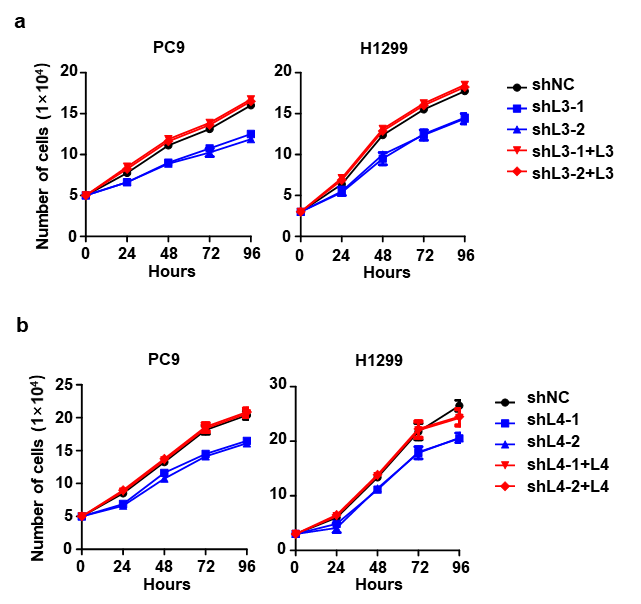


**Supplementary Figure 2. Rescue assays in NSCLC cells after stable silencing of lncRNA LCETRL3 or LCETRL4 with shRNAs.** (a) Over-expression of LCETRL3 could enhance cell proliferation of NSCLC cells after stable silencing of lncRNA LCETRL3 with shRNAs. (b) Over-expressed LCETRL4 enhance cell growth of NSCLC cells after stable knocking-down of lncRNA LCETRL4 with shRNAs.


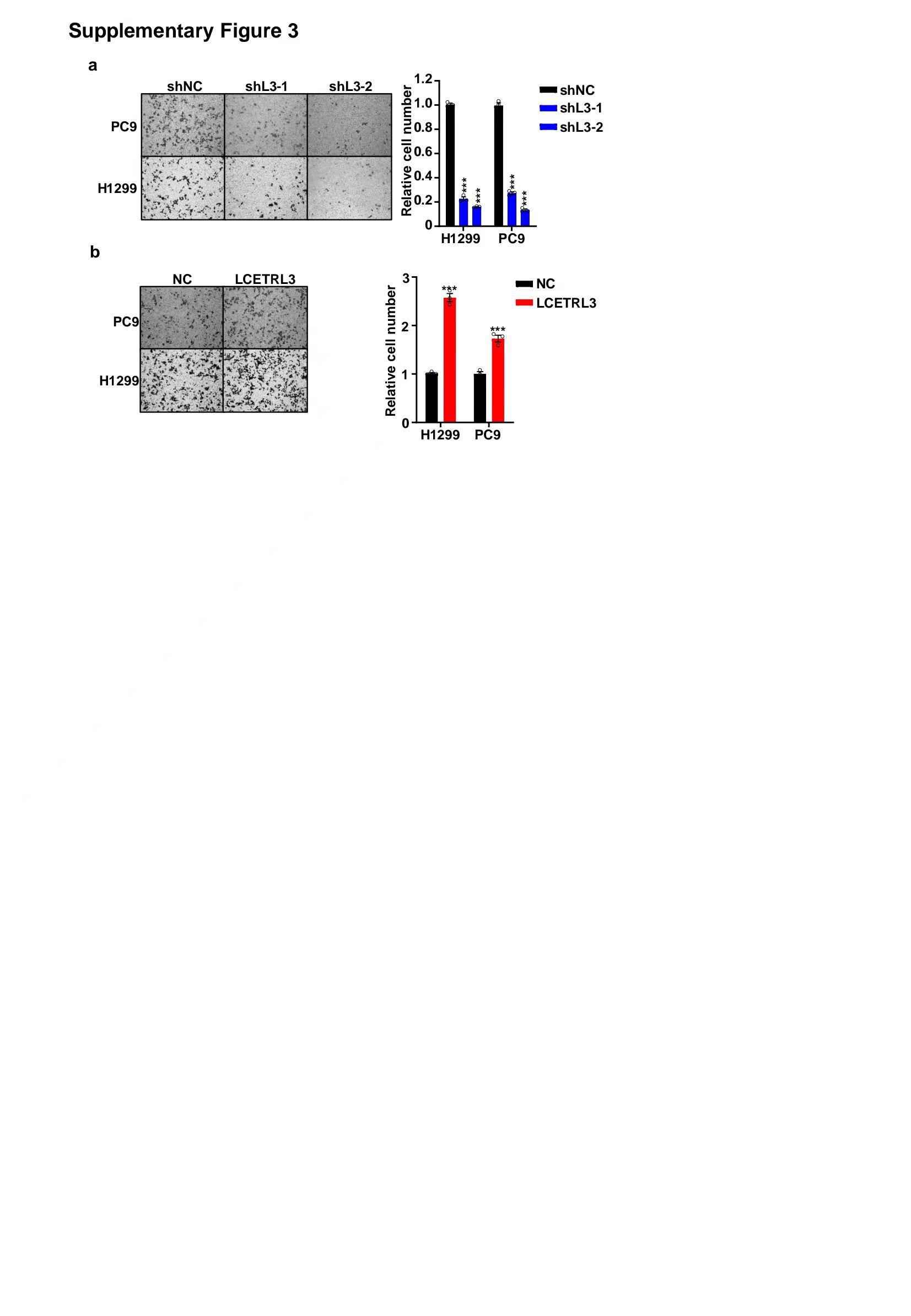


**Supplementary Figure 3. LncRNA LCETRL3 promotes invasion of NSCLC cells.** Matrigel transwell assays indicated that LCETRL3 promoted invasion of NSCLC cells (a, b).


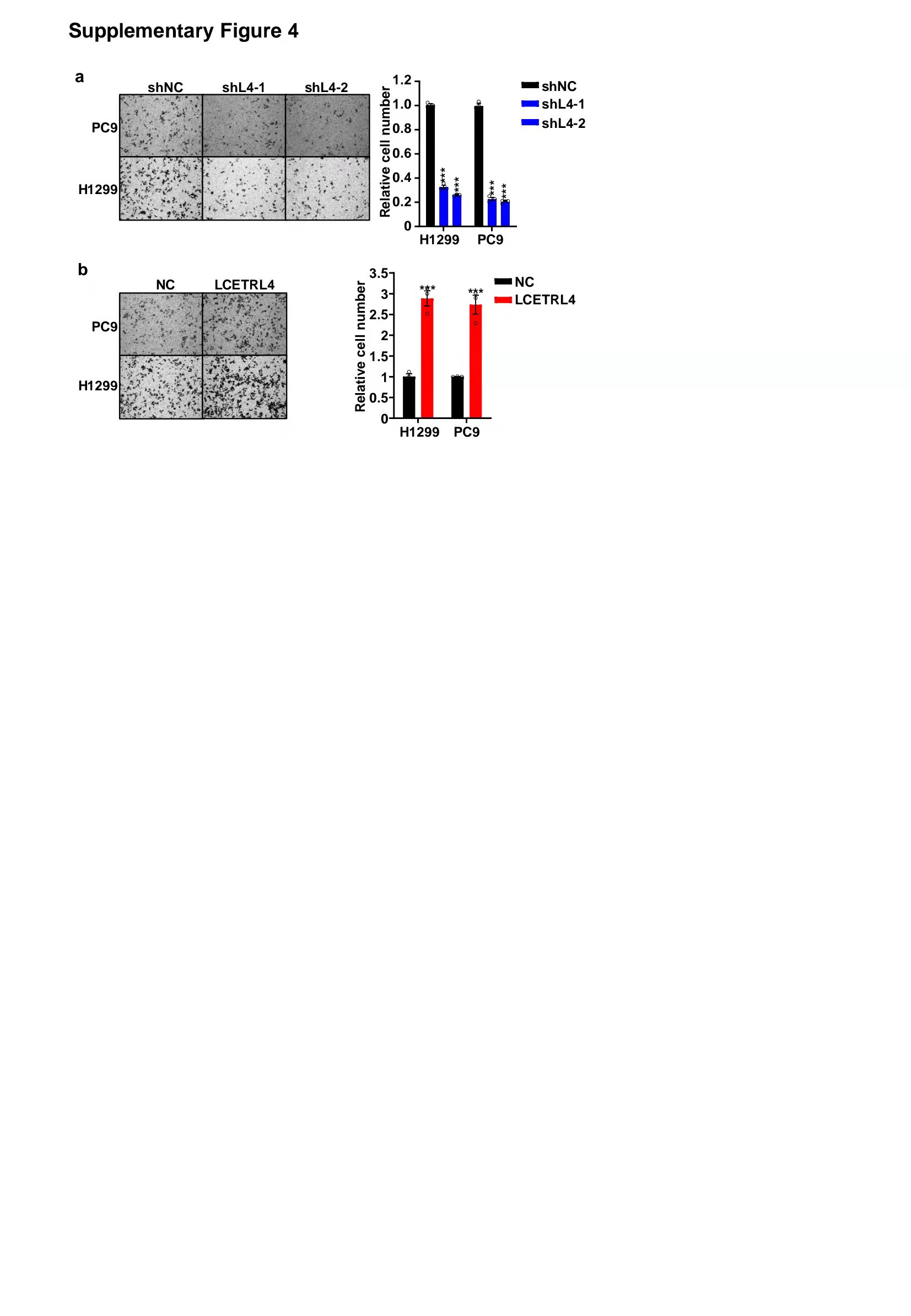


**Supplementary Figure 4. LncRNA LCETRL4 stimulates invasion of NSCLC cells.** Matrigel transwell assays indicated that LCETRL4 promoted invasion of NSCLC cells (a, b).


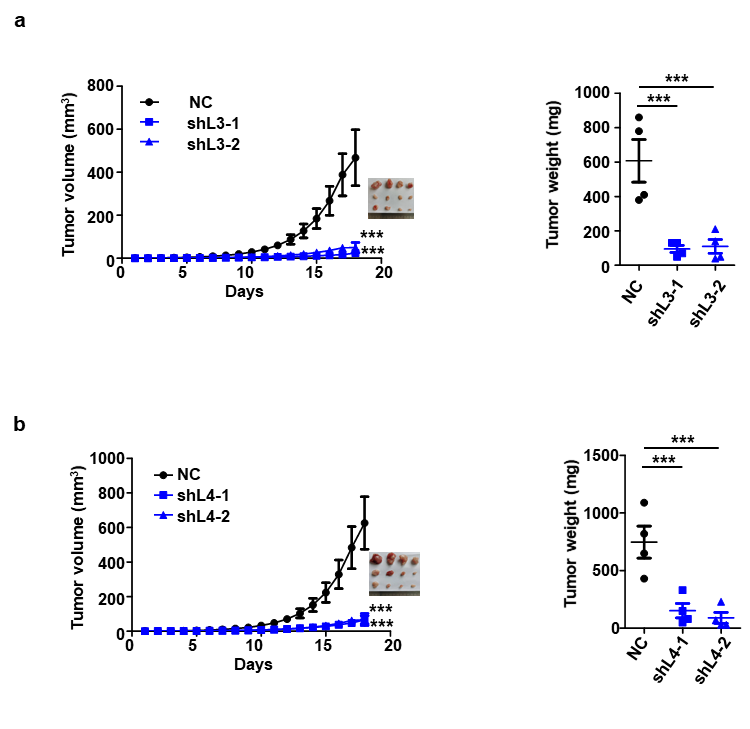


**Supplementary Figure 5. Silencing of lncRNA LCETRL3 or LCETRL4 inhibits proliferation of NSCLC cells *in vivo*.** Stable knocking-down of LCETRL3 or LCETRL4 evidently inhibited growth of the NSCLC xenografts compared to the control xenografts (a, b)


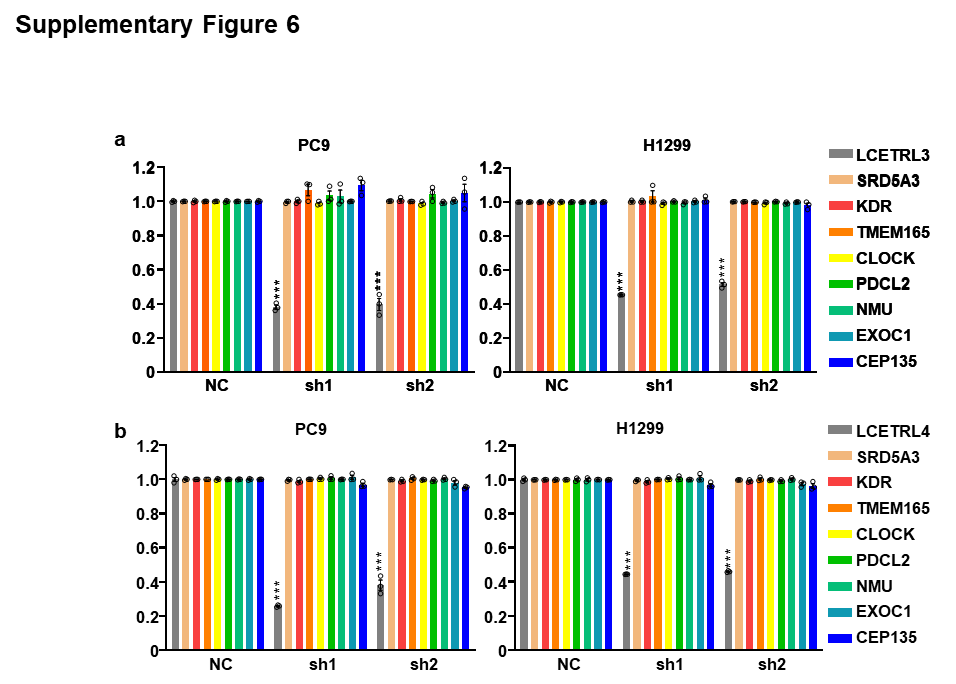


**Supplementary Figure 6. LCETRL3 or LCETRL4 shows no impacts on expression of adjacent protein coding genes at 4q12.** Relative mRNA expression of adjacent protein coding genes at 4q12 after silencing of lncRNA LCETRL3 (a) or LCETRL4 (b).


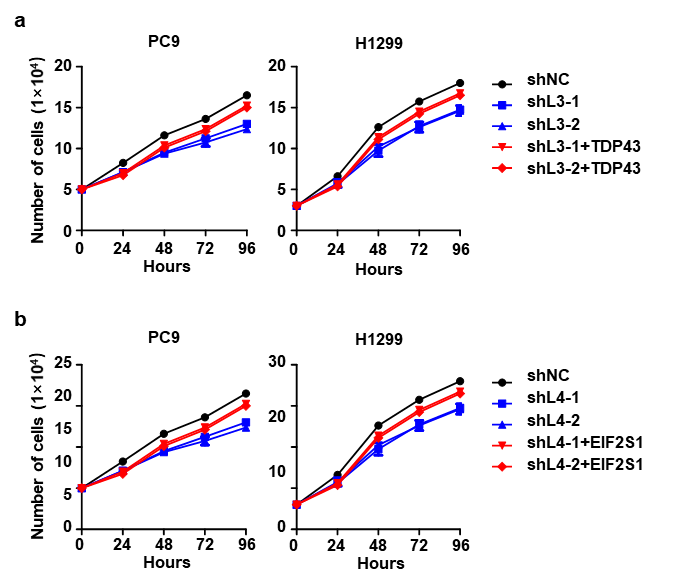


**Supplementary Figure 7. Over-expression of TDP43 or EIF2S1 in NSCLC cells after stable silencing of lncRNA LCETRL3 or LCETRL4 with shRNAs.** (a) Over-expression of TDP43 could promote cell viability of NSCLC cells after stable silencing of lncRNA LCETRL3 with shRNAs. (b) Over-expressed EIF2S1 could promote cell growth of NSCLC cells after stable knocking-down of lncRNA LCETRL3 with shRNAs.

**Supplementary Table 1. Five candidate lncRNAs at 4q12.**

| **LncRNA names** | **Ensemble names** | **Ensemble IDs** |
| --- | --- | --- |
| LCETRL1 | RP11-530I17.2 | ENST00000507445.2 |
| LCETRL2 | SRD5A3-AS1 | ENST00000510637.1 |
| LCETRL3 | RP11-177J6.1 | ENST00000609511.1 |
| LCETRL4 | RP11-528I4.2 | ENST00000609234.1 |
| LCETRL5 | RP11-345F18.1 | ENST00000504250.1 |

**Supplementary Table 2. Mass spectrometry of proteins pulled-down by LCETRL3 in PC9 and H1299 cells**

| **No.** | **Protein names** | **Gene names** | **LFQ intensity in PC9** | | **LFQ intensity in H1299** | | **Proteins** | **Peptides** |
| --- | --- | --- | --- | --- | --- | --- | --- | --- |
|  |  |  | **Sense** | **Antisense** | **Sense** | **Antisense** |  |  |
| **1** | **TAR DNA-binding protein 43** | **TDP43** | **31725000** | **0** | **14515000** | **0** | **19** | **3** |
| 2 | Inosine-5-monophosphate dehydrogenase 2 | IMPDH2 | 19443000 | 0 | 10153000 | 0 | 5 | 2 |
| 3 | Ubiquitin carboxyl-terminal hydrolase 10 | USP10 | 35255000 | 0 | 0 | 0 | 4 | 2 |
| 4 | Cell division cycle 5-like protein | CDC5L | 70412000 | 0 | 8393000 | 0 | 3 | 1 |
| 5 | Signal recognition particle receptor subunit alpha | SRPR | 24292000 | 0 | 45902000 | 0 | 3 | 3 |
| 6 | DnaJ homolog subfamily C member 9 | DNAJC9 | 0 | 0 | 6071600 | 0 | 3 | 2 |
| 7 | ATP-dependent RNA helicase DHX29 | DHX29 | 94986000 | 0 | 12763000 | 0 | 2 | 3 |
| 8 | Prolactin-inducible protein | PIP | 1.05E+08 | 0 | 1.13E+08 | 0 | 1 | 3 |

**Supplementary Table 3. Mass spectrometry of proteins pulled-down by LCETRL4 in PC9 and H1299 cells**

| **No.** | **Protein names** | **Gene names** | **LFQ intensity in PC9** | | **LFQ intensity in H1299** | | **Peptides** | **Unique** |
| --- | --- | --- | --- | --- | --- | --- | --- | --- |
|  |  |  | **Sense** | **Antisense** | **Sense** | **Antisense** |  |  |
| **1** | **Eukaryotic translation initiation factor 2 subunit 1** | **EIF2S1** | **2371900** | **0** | **3429700** | **0** | **2** | **2** |
| 2 | Enhancer of rudimentary homolog | ERH | 0 | 0 | 3729900 | 0 | 1 | 1 |
| 3 | SRA stem-loop-interacting RNA-binding protein, mitochondrial | SLIRP | 1702500 | 0 | 851590 | 0 | 1 | 1 |
| 4 | Histone deacetylase complex subunit SAP30 | SAP30 | 10362000 | 0 | 19529000 | 0 | 1 | 1 |

**Supplementary Table 4. Basic characteristics of the two cohorts in this study.**

| **Cohorts** | **Discovery(n=20)** | **Validation(n=44)** |
| --- | --- | --- |
| Age-no. (%) |  |  |
| ＞60 | 10(50%) | 20(45.4%) |
| ≤60 | 10(50%) | 24(54.6%) |
| Sex-no. (%) |  |  |
| Male | 15(75%) | 29(65.9%) |
| Female | 5(25%) | 15(34.1%) |
| Stage-no. (%) |  |  |
| Ⅰ&Ⅱ | 12(60%) | 24(54.5%) |
| Ⅲ | 8(40%) | 20(45.5%) |
| Pathologic types-no. (%) |  |  |
| LUSC | 10(50%) | 19(43.2%) |
| LUAD | 10(50%) | 25(56.8%) |

**Supplementary Table 5. Primers for RT-qPCR**

| Name | Primer Sequence |
| --- | --- |
| ß-ACTIN-qF | GGCGGCACCACCATGTACCCT |
| ß-ACTIN-qR | AGGGGCCGGACTCGTCATACT |
| LCETRL1-qF | AGATTCTCAAGTTGAGAGCT |
| LCETRL1-qR | GGGATTGTAATTCATTCACCAC |
| LCETRL2-qF | CCCAGACAACTTGAAGAAGC |
| LCETRL2-qR | ATGCTCCTTATACACATCAGTC |
| LCETRL3-qF | CCTACCCTCTCAACGACAGC |
| LCETRL3-qR | CTCTGACCTTTTGCCAGGAG |
| LCETRL4-qF | TAACATCTTCTCCCATCTCC |
| LCETRL4-qR | CATTCTCCATCTTCCATTACAC |
| LCETRL5-qF | CACCCATTATTGTCTTTGGCT |
| LCETRL5-qR | ATTCCATACCCACCCTTTCC |
| S14-qF | GGCAGACCGAGATGAATCCTC |
| S14-qR | CAGGTCCAGGGGTCTTGGTCC |
| U2-qF | CATCGCTTCTCGGCCTTTTG |
| U2-qR | TGGAGGTACTGCAATACCAGG |
| NMR-qF | CCGTTTGCTGAAGCCGAAGA |
| NMR-qR | GCGGTGGGTGGCGTGAAGT |
| FTX-qF | AGTTCAGGTCATCTCCACAC |
| FTX-qR | CCAATTATGCCCACTCTCAG |
| CEP135-qF | ATAGATCAGTTAGCACAGCAG |
| CEP135-qR | GATGAACAACAGTTTCTAGGTC |
| CLOCK-qF | GTCAGCAAACATCTCTACCC |
| CLOCK-qR | AATCTTATCTGCCTGTCCTGAG |
| NMU-qF | GCTACAGTTGTGGAATGAGG |
| NMU-qR | ATTTGACTTGCCCAACTTCTG |
| PDCL2-qF | GCTACAGTTGTGGAATGAGG |
| PDCL2-qR | ATTTGACTTGCCCAACTTCTG |
| SRD5A3-qF | GCCTTACTCAATCTCTGTTCC |
| SRD5A3-qR | CAGCCACAGAAATACTAGCA |
| TMEM165-qF | TTAAAGATGAGCCCTGATGAG |
| TMEM165-qR | TTGTAGTTAGTTGAGAGCGA |
| EXOC1-qF | TCCTTCTGACCTTCCTTTCC |
| EXOC1-qR | TGACTTTGACCACCTTAACC |
| GRB2-qF | AGAAATGAAACCACATCCGT |
| GRB2-qR | TCTGTGATAATCCACCAGCTC |
| KDR-qF | GGATGAACATTGTGAACGAC |
| KDR-qR | CATCTGCTTCAATCACTTGG |
| EIF2S1-F | GAAGGCGTATCCGTTCTATCAAC |
| EIF2S1-R | AGCAACATGACGAAGAATGCTAT |
| TDP43-F | GGGTAACCGAAGATGAGAACG |
| TDP43-R | CTGGGCTGTAACCGTGGAG |

**Supplementary Table 6. Sequences of siRNAs**

| Name | Sense (5’-3’) | Antisense (5’-3’) |
| --- | --- | --- |
| siL3-1 | CCAGGACCCUAAUUUGCAUTT | AUGCAAAUUAGGGUCCUGGTT |
| siL3-2 | GAGAUCUUUACAGUCUUAATT | UUAAGACUGUAAAGAUCUCTT |
| siL4-1 | CCUGGGUUGAAGGAGUCUUTT | AAGACUCCUUCAACCCAGGTT |
| siL4-2 | GGAAAGGGUGGGUAUGGAATT | UUCCAUACCCACCCUUUCCTT |

**Supplementary Table 7. Sequences of shRNAs**

| Name | shRNA Sequence |
| --- | --- |
| shLCETRL3-1 | CGGCCTGGGTTGAAGGAGTCTTCTCGAGAAGACTCCTTCAACCCAGGTTTTT |
| shLCETRL3-2 | CGGGGAAAGGGTGGGTATGGAACTCGAGTTCCATACCCACCCTTTCCTTTTT |
| shLCETRL4-1 | CGGCCAGGACCCTAATTTGCATCTCGAGATGCAAATTAGGGTCCTGGTTTTT |
| shLCETRL4-2 | CGGGAGATCTTTACAGTCTTAACTCGAGTTAAGACTGTAAAGATCTCTTTTT |
| shNC | CGGTAGTCGCATACGGAACATTCGCTCGAGCGAATGTTCCGTATGCGACTATTTTT |

**Supplementary Table 8. Antibodies used in the study**

| Name | Company | Catalog Number |
| --- | --- | --- |
| TDP43 | PTG | 10782-2-AP |
| EIF2S1 | PTG | 11170-1-AP |
| CDCL5 | Abcam | ab129114 |
| DHX29 | PTG | 13923-1-AP |
| p-AKT (Thr308) | CST | 13038T |
| p-AKT (Ser473) | CST | 40605 |
| AKT | Abcam | ab179463 |
| IMPDH2 | Abcam | ab131158 |
| PTEN | Abcam | ab267787 |
| NOTCH1 | Abcam | ab52627 |
| p-PDK1 (Ser241) | CST | 3438T |
